# Risk of Bias Evaluation of Cross‐Sectional Studies: Adaptation of the Newcastle‐Ottawa Scale

**DOI:** 10.1111/jre.13405

**Published:** 2025-04-28

**Authors:** Maria Clotilde Carra, Pierluigi Romandini, Mario Romandini

**Affiliations:** ^1^ Department of Translational Medicine University of Ferrara Ferrara Italy; ^2^ METHODS Team, CRESS, INSERM, INRAe Université Paris Cité Paris France; ^3^ Division of Fixed Prosthodontics and Biomaterials University Clinics for Dental Medicine, University of Geneva Geneva Switzerland; ^4^ Perio‐Implant Innovation Center, Institute for Integrated Oral, Craniofacial and Sensory Research—National Clinical Research Center of Stomatology Ninth People's Hospital, Shanghai Jiao Tong University School of Medicine Shanghai China

**Keywords:** bias, cross‐sectional studies, epidemiologic factors, epidemiology, methods, prevalence, risk factors, systematic review

## Abstract

Cross‐sectional studies are widely utilized in medical research to estimate prevalence and examine associations. As such, they can serve as a significant source of data for systematic reviews. However, specific considerations are necessary when evaluating the risk of bias (RoB) of cross‐sectional studies, as several potential biases can undermine the validity, reliability, and robustness of their findings. This article introduces a novel, context‐specific tool designed to assess the RoB of cross‐sectional studies for use in systematic reviews. The proposed tool represents an adaptation of the Newcastle‐Ottawa Scale (NOS), originally developed for cohort and case–control studies. Similar to the original NOS, the new tool (named “NOS‐xs”) features a nine‐star rating system to assess six specific items across three main domains: (i) study sample selection, (ii) assessment of exposure(s) and outcome(s), and (iii) confounding factors. Based on the number of awarded stars, studies are categorized as having high (0–3 stars), moderate (4–6 stars), or low (7–9 stars) RoB. The NOS‐xs tool maintains consistency with the original NOS tool, facilitating its integration into systematic reviews that also include cohort and/or case–control studies. While the NOS‐xs is suited to analytical cross‐sectional studies (i.e., association studies), a simplified version (“NOS‐xs2”) is also introduced for descriptive cross‐sectional studies (i.e., prevalence studies). The NOS‐xs2 features a four‐star rating system to assess three of the six specific items included in the NOS‐xs. To streamline their application, spreadsheets for both NOS‐xs and NOS‐x2 are provided.

## Graphical abstract

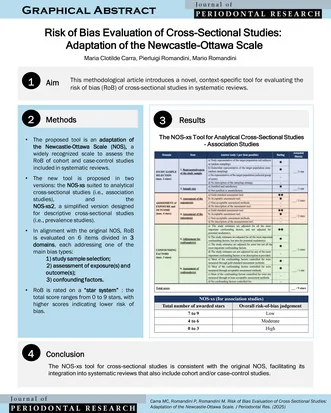

## 1 Introduction

Risk of bias (RoB) assessment is a critical component of systematic reviews [1, 2]. By assessing RoB, reviewers can evaluate the quality of individual studies and interpret their findings accurately. Specific RoB tools have been developed for various study designs. For instance, the RoB‐II tool is the Cochrane Collaboration's recommended instrument for appraising randomized clinical trials [3], while ROBINS‐I is preferred for non‐randomized controlled clinical trials [2, 4]. For cohort and case–control studies, the Newcastle Ottawa Scale (NOS) is the most commonly used tool [5, 6, 7]. However, no consensus exists on the optimal instrument for assessing RoB in cross‐sectional studies. Although several tools have been used or adapted for this purpose, none comprehensively address the range of biases inherent to this study design [6].

This methodological article introduces a novel, context‐specific tool for evaluating the RoB of cross‐sectional studies in systematic reviews. The proposed tool, named NOS‐xs, is an adaptation of the NOS scale originally developed for cohort and case–control studies. While the full version of the NOS‐xs is suited to analytical cross‐sectional studies (i.e., association studies), a simplified version (NOS‐xs2) is also provided for descriptive ones (i.e., prevalence studies).

### 1.1 Context Definition: Cross‐Sectional Studies

In clinical research, cross‐sectional studies are highly popular. They provide valuable insights on the prevalence of diseases/conditions, as well as on associations of variables of interest at a specific point in time. When used for analytical purposes—specifically to examine associations between two variables, an exposure (typically a putative risk factor/risk indicator) and an outcome (usually a disease or condition)—care must be taken to avoid inferring causality. This is because cross‐sectional studies cannot establish temporality (i.e., whether the exposure precedes the outcome) and inherently carry risks of residual confounding and reverse causation [8].

Due to their observational nature and lack of a temporal dimension, cross‐sectional studies are therefore primarily suited for assessing prevalence. However, they can also serve as a preliminary step in hypothesis generation and in evaluating the need for future longitudinal or interventional studies to confirm temporal and causal relationships [8, 9]. Cross‐sectional studies offer indeed several advantages as first‐line approaches for exploring associations. They represent the simplest individual‐level observational study design and are generally less expensive, resource‐efficient, ethically acceptable, and easier to conduct compared to case–control or cohort studies. Despite these advantages, cross‐sectional studies are inherently limited by potential biases that are not always effectively controlled or addressed [1, 10].

### 1.2 Types of Bias in Cross‐Sectional Studies

Cross‐sectional studies are susceptible to several types of bias that may arise during the population selection or sampling process (i.e., selection bias), the ascertainment of exposure(s) and outcome(s) (i.e., information bias), and the influence of confounders (i.e., confounding bias) [2, 6].

Cross‐sectional studies can be either descriptive (e.g., prevalence studies) or analytical (e.g., association studies). While all the aforementioned bias types are relevant to analytical studies, biases related to the assessment of exposure and the influence of confounders are not applicable to descriptive studies [11].

## 2 NOS‐xs: Adaptation of the Newcastle‐Ottawa Scale for Cross‐Sectional Studies

The NOS was originally developed to assess the quality of case–control and cohort studies included in systematic reviews [5]. Created by researchers from the Universities of Newcastle (Australia) and Ottawa (Canada), the NOS uses a “star system” to evaluate studies across three domains: selection of the study groups, comparability of the groups, and ascertainment of either the exposure (for case–control studies) or the outcome (for cohort studies). However, applying the NOS directly to cross‐sectional studies is not feasible without omitting certain items (e.g., selection of controls or follow‐up adequacy) or altering their intended interpretation.

To align with the structure of the original tool, we propose the NOS‐xs using three domains, each addressing one of the main bias types:

- Study sample selection;
- Assessment of exposure(s) and outcome(s);
- Confounding factors.

The RoB is then assessed across 6 items (two per domain), each with predefined answers corresponding to ratings based on the “star system”. Only one answer can be selected per item, and stars are awarded for high quality choices. The NOS‐xs is presented in Table 1, with an editable version available as Supporting Information–S1. The maximum number of stars per domain is as follows:

- Study sample selection: 2 stars,
- Assessment of exposure(s) and outcome(s): 4 stars,
- Confounding factors: 3 stars.

**TABLE 1:** NOS‐xs: adaptation of the NOS for cross‐sectional studies – association studies.

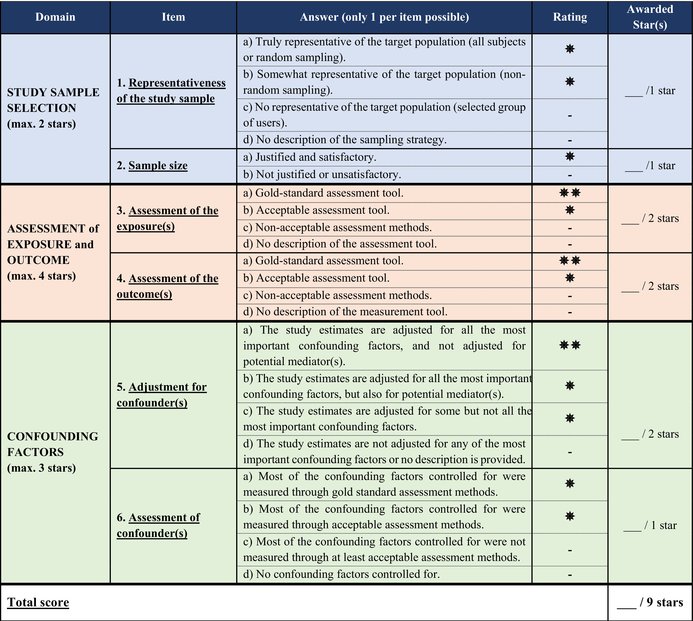

To maintain consistency with the original NOS, the number of stars awarded per domain and the weighting of individual items (1 or 2 stars) have been preserved whenever possible. This results in a total score ranging from 0 to 9 stars, with a higher score indicating a lower RoB.

As in the original tool, the overall RoB is finally categorized as follows:

- Low RoB: 7–9 stars;
- Moderate RoB: 4–6 stars;
- High RoB: 0–3 stars.

### 2.1 Study Sample Selection

This domain is evaluated across two items:

1. Representativeness of the sample (maximum: one star);
2. Sample size (maximum: one star).

Selection bias is minimized when the study sample accurately represents the target population. This can be achieved by including all population subjects or employing random sampling. However, a non‐randomly sampled population that still aligns closely with the target population can be deemed somewhat representative and awarded a star. The representativeness of the sample is critical for the generalizability of the findings, as unrepresentative samples compromise external validity and hinder the applicability of results beyond the study context.

The sample size must be justified and sufficient to assess the association between exposure(s) and outcome(s), while properly accounting for potential confounders in the statistical analysis. This should be demonstrated through either a priori sample size calculation or, at a minimum, a post hoc power analysis specific to the study's objectives.

### 2.2 Assessment of Exposure(s) and Outcome(s)

This domain is evaluated across two items:

1. Assessment of the exposure(s) (maximum: two stars);
2. Assessment of the outcome(s) (maximum: two stars).

Studies employing “gold standard” assessment methods for the specific exposure(s) and outcome(s) are awarded two stars. Studies using alternative, “acceptable” and validated tools are awarded one star. This approach minimizes information bias (e.g., observer, interviewer, recall, or detection biases), and ensures the validity and reliability of the measured variables.

The definition of “gold standard” and “acceptable” alternative methods to measure the exposure(s) and the outcome(s) must occur a priori, during systematic review protocol preparation, before the RoB evaluation begins. Depending on the focused question of the systematic review, the appropriateness of specific assessment methods may indeed vary. These may include specific case definitions assessed through different combinations of clinical and/or radiographic parameters, self‐reporting, or database records. Depending on the systematic review and its context, additional methodological considerations, such as examiner calibration or blinding, may be additionally warranted.

### 2.3 Confounding Factors

This domain is evaluated across two items:

1. Adjustment for confounder(s) (maximum: two stars);
2. Assessment of confounder(s) (maximum: one star).

A confounder is a variable that influences the association between an exposure and an outcome. Proper identification and adjustment for confounders are essential to ensure the validity of findings derived from cross‐sectional studies.

The first item assesses whether the study considered the role of potential confounders in the statistical analyses. In the former case, the study may control for all the “most important confounders”. If these adjustments are considered satisfactory and no adjustment is performed for “possible mediators” (i.e., variables involved in the possible causal pathway between the two variables), two stars can be awarded. If the main estimates are only adjusted for some of the “most important confounders” or adjusted for “possible mediators”, only one star is awarded. When no confounder is considered or no description is provided, no star is awarded. The list of “most important confounders” and “possible mediators” must be explicitly pre‐determined at the time of systematic review protocol preparation. If a study reports more than one estimate of association (e.g., both crude and adjusted models), the assessment is performed on the estimate of interest (i.e., the “best” model in terms of adjustment). Additionally, the systematic review protocol should specify whether certain statistical tests, such as stratification or propensity score matching, are acceptable substitutes for full adjustment. It should also explicitly state whether deviations from predefined lists of confounders and mediators are permitted, provided they are justified by statistical testing (e.g., demonstrating no evidence of confounding through the “change‐in‐estimate” strategy [12, 13], or ruling out mediation effects [14, 15]).

The second item evaluates the validity of the methods used to measure the confounding factors. If “gold standard” or “acceptable” methods are mostly used, the study can be awarded one star.

## 3 NOS‐xs2: Simplified Version for Prevalence Studies

Since biases related to the assessment of exposure and the influence of confounders are not applicable to descriptive studies (i.e., prevalence studies), a simplified version of the NOS‐xs, named NOS‐xs2, is proposed to evaluate their RoB. This simplified version assesses RoB using two domains:

- Study sample selection;
- Assessment of outcome(s);

RoB is evaluated across three items: the same two of NOS‐xs for “study sample selection” and the item for “assessment of outcome(s)”. Each item keeps the same predefined answers aligned with the “star system”, awarding stars for high‐quality choices. Only one answer per item can be selected. The NOS‐xs2 is presented in Table 2, with an editable version available as Supporting Information S2. The maximum number of stars for each domain is as follows:

- Study sample selection: 2 stars,
- Assessment of outcome(s): 2 stars.

**TABLE 2:** NOS‐xs2: simplified version for cross‐sectional prevalence studies.

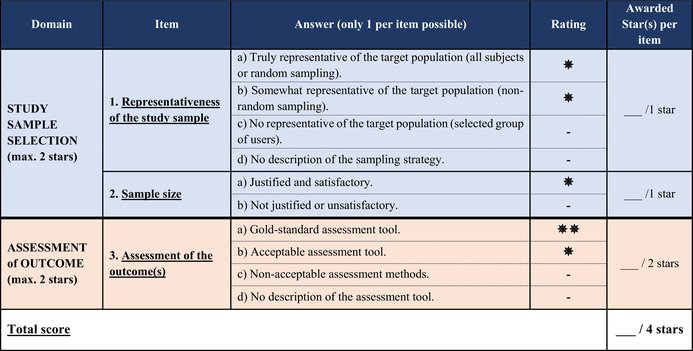

Given the reduced number of items, the total score ranges from 0 to 4 stars. The overall interpretation of RoB still corresponds to the total number of stars, categorized as follows (Table 3):

- Low RoB: 3–4 stars;
- Moderate RoB: 2 stars;
- High RoB: 0–1 star.

**TABLE 3:** Interpretation of the NOS‐xs and NOSxs2.

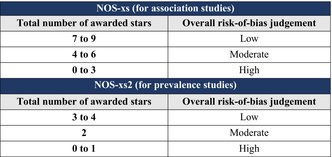

## 4 Customizing NOS‐xs and NOS‐xs2 in Systematic Review Protocols

Based on the specific focus and scope of the systematic review, the NOS‐xs and NOS‐xs2 necessitate careful customization to align with the characteristics of the studies being evaluated. This customization must be performed a priori, during the systematic review protocol preparation phase. For study sample selection, customization involves defining the target population in detail, such as the geographic region or specific settings relevant to the review question. For the assessment of exposure and outcomes, the protocol must specify the “gold standard” and “acceptable” methods for exposure and outcome assessment, as well as non‐acceptable methods. It should also define whether calibration and/or blinding of assessors are mandatory. For confounding factors, customization involves identifying the “most important” confounders and “possible” mediators relevant to the review's research question. The protocol should also specify whether certain statistical methods, such as propensity score matching, are acceptable alternatives to direct adjustments for confounders. Additionally, it should address whether deviations from the predefined lists of confounders or mediators are permissible, provided they are justified by statistical testing. Finally, the protocol must specify the “gold standard” and “acceptable” methods for confounders assessment.

A checklist to guide this customization process is provided in Table 4, with editable versions for NOS‐xs and NOS‐x2 available as Supporting Information S3 and S4, respectively. It is recommended to include the appropriate checklist as an attachment to the systematic review protocol.

**TABLE 4:** Checklist to customize the NOS‐xs during systematic review protocol preparation.

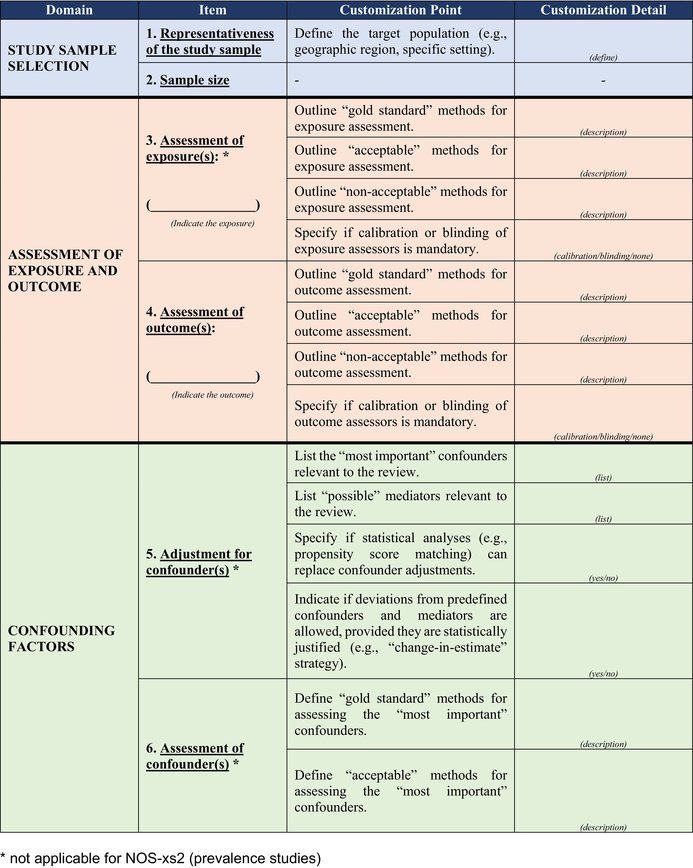

## 5 NOS‐xs Spreadsheets and Case Examples of Application

To facilitate the use of the NOS‐xs and NOS‐xs2 in assessing the RoB in cross‐sectional studies, a dedicated Excel spreadsheet is also provided (Supporting Information S5). This tool allows reviewers to evaluate each item using a drop‐down menu, automatically assigning the corresponding star(s) and calculating the final score. Alternatively, reviewers can use an editable PDF version of NOS‐xs (Supporting Information S6) or NOS‐xs2 (Supporting Information S7).

To guide readers, two case examples demonstrate the application of NOS‐xs and NOS‐xs2, drawing from articles published in the *Journal of Periodontal Research*.

### 5.1 Application of NOS‐xs to an Association Study

Molina et al. [16] investigated the association between periodontitis and the Systematic Coronary Risk Evaluation (SCORE) algorithm in Spanish employed adults participating in the Workers' Oral Health study.

#### 5.1.1 Domain 1, Item 1 (Study Sample Selection: Representativeness)

The study was awarded 1 star for item 1 (answer “a”). This was based on the study's representativeness of the Spanish working adult population aged 16–70 years, achieved through the following random sampling method:

A proportionate stratified random sampling method was applied, using age, sex, and geographical area of the participants.

#### 5.1.2 Domain 1, Item 2 (Study Sample Selection: Sample Size)

For item 2, 0 out of 1 star was awarded (answer “b”) because the study lacked a justification for the final sample size, with no a priori sample size calculation or *post hoc* power analysis specific to the study's objectives.

#### 5.1.3 Domain 2, Item 3 (Assessment of Exposure)

The exposure, periodontitis, was assessed by trained and calibrated examiners using clinical attachment level (CAL) measurements at 10 index teeth:

The periodontal examination consisted of the evaluation of their periodontal affectation using […] CAL at 10 index teeth (1.7, 1.6, 1.1, 2.6, 2.7, 3.7, 3.6, 3.1, 4.6, and 4.7). […] According to their CAL levels (0–3 mm, 4–5 mm, and ≥ 6 mm), participants were categorized dichotomously as CAL 0–3 mm versus ≥ 4 mm, and 0–5 mm versus ≥ 6 mm.

Hypothesizing the method was deemed “acceptable” rather than “gold standard” during the protocol preparation phase of the systematic review (given the validation of CAL use for periodontitis assessment but limited by partial mouth recording), 1 out of 2 stars was awarded (answer “b”). This case definition could indeed potentially bias prevalence estimates by under‐ or overestimating disease presence.

#### 5.1.4 Domain 2, Item 4 (Assessment of Outcome)

The outcome, cardiovascular risk based on the SCORE algorithm, was assessed as follows:

CVD risk was calculated for each subject according to the European SCORE model for fatal vascular events in low‐risk countries. Subjects with CVD, type 2 diabetes, systolic blood pressure ≥ 180 mmHg or diastolic blood pressure ≥ 110 mmHg, total cholesterol > 320 mg/dL, LDL cholesterol > 240 mg/dL, and those with relative risk (RR) > 4 were considered as having high risk. Patients were grouped into three categories of CVD risk, according to their SCORE: low (SCORE < 1%), moderate (SCORE 1%–4%), and high/very high risk (SCORE ≥ 5%).

The algorithm used was not the updated “SCORE 2” model [17]. Therefore, assuming this method was considered “acceptable” but not “gold standard” during the protocol preparation phase of the systematic review, only one star can be awarded for this item as well (answer “b”).

#### 5.1.5 Domain 3, Item 5 (Confounding Factors: Adjustment)

The study described the following adjustments:

[…] logistic regressions […] after considering adjustment for the following confounding factors: age, sex, smoking habit, glycemic levels, education level, occupation, and toothbrushing frequency (explanatory regression). Selection of the confounders was made following a theoretical criterion of plausibility […] and upon the significance of covariates in the univariate analysis with both the exposure and the outcome (*p* < 0.20). […] In order to select the best reduced model, the “Confound” command of STATA was used to select the best reduced model according to Hosmer–Lemeshow. Only those confounders that produce a clinically significant change in the odds ratio (OR) (10%), with the highest precision based on their 95% CI, were included in the adjusted model.

Assuming the systematic review protocol defined smoking, glycemic status, and socioeconomic status as the “most important” confounders for this association, C‐reactive protein as the “possible mediator”, and permitted deviations from the predefined lists of confounders if justified by the “change‐in‐estimate” strategy, this study considered all three “most important” confounders. Moreover, they were included in each actual model based on the “change‐in‐estimate” strategy and did not adjust for the “possible mediator” defined a priori. Therefore, two stars are awarded for item 5 (answer “a”).

#### 5.1.6 Domain 3, Item 6 (Confounding Factors: Assessment)

As described in the Methods section of the publication, most of the confounding factors considered were measured using acceptable assessment methods, even if they were not always the gold standard (item 6, answer “b”). Consequently, 1 star is assigned.

#### 5.1.7 Overall RoB

Summing the awarded stars, Molina et al. [16] scored 6 out of a maximum of 9 stars:

– Study sample selection: 1 out of 2
– Assessment of exposure and outcome: 2 out of 4
– Confounding factors: 3 out of 3

This total score places the study at a moderate RoB according to the NOS‐xs system.

### 5.2 Application of NOS‐xs2 to a Prevalence Study

Matarazzo et al. 2018 [18] analyzed the prevalence of peri‐implant diseases in a Brazilian university‐based population.

#### 5.2.1 Domain 1, Item 1 (Study Sample Selection: Representativeness)

The study population consisted of a convenience sample of patients:

The study population was composed by individuals rehabilitated with implant‐supported fixed prosthesis treated at the Dental Clinic of the State University of Maringá, Brazil, between 2004. And 2010. Patients with one or more dental implants in masticatory function for at least 1 year were contacted and invited to return to the Dental Clinic to be clinically and radiographically examined.[…] Of the 281 subjects treated in that period, a total of 211 individuals (75% of all patients treated) participated in the study.

The selected population cannot be considered representative of the average target population in Brazil due to its specific geographical provenance and setting (university clinic). Additionally, as is typical for convenience samples of patients with dental implants, non‐responders (25%) are likely to differ substantially from responders in terms of peri‐implant diseases prevalence [19, 20, 21]. Indeed, non‐responders often did not receive preventive measures (i.e., structured supportive peri‐implant care) and typically belong to a socioeconomic context more prone to disease. Therefore, 0 star are awarded (answer “c”).

#### 5.2.2 Domain 1, Item 2 (Study Sample Selection: Sample Size)

For item 2, 0 out of 1 star was awarded (answer “b”) because the study lacked justification for the final sample size, with no a priori sample size calculation or *post hoc* power analysis specific to the study's objectives.

#### 5.2.3 Domain 2, Item 3 (Assessment of Outcome)

As indicated in the Methods section of the publication, the outcomes (i.e., peri‐implant mucositis and peri‐implantitis) were assessed by calibrated examiners. However, given the absence of radiographic documentation at the time of implant functional loading, bone loss was evaluated indirectly [22]. This case definition, although acceptable and validated, has proven to be less accurate than case definitions employing baseline documentation [23]. Thus, only 1 star can be awarded (answer “b”).

#### 5.2.4 Overall RoB

Summing the awarded stars, the study by Matarazzo et al. [18] scored 1 out of a maximum of 4 stars:

– Study sample selection: 0 out of 2
– Assessment of outcome: 1 out of 2

This total score places the study at a high RoB according to the NOS‐xs2 system.

## 6 Discussion

This methodological article introduces the NOS‐xs tool, a tailored scale for evaluating the RoB in cross‐sectional studies, designed for application in systematic reviews. Adapted from the NOS developed for cohort and case–control studies, the NOS‐xs employs the same star‐based system of the original tool across six targeted items. The alignment with the original NOS aims to facilitate its integration into systematic reviews that also include cohort and/or case–control studies. Moreover, the NOS‐xs retains the main characteristics of the original tool, being user‐friendly, time‐efficient, and widely recognized.

While the full version of the NOS‐xs is suited to analytical cross‐sectional studies (Table 1), a simplified version, namely NOS‐xs2, is also provided for descriptive ones (Table 2). This latter version was created to avoid further non‐standardized adaptations of the tool that would have been necessary for systematic reviews including cross‐sectional studies solely to estimate disease prevalence. Additionally, Excel spreadsheets and editable PDF files of the NOS‐xs and NOS‐xs2 tools are provided as supplementary materials to streamline their application in future systematic reviews.

In medical research, cross‐sectional studies are a widely used form of observational design, yet they possess distinct characteristics that require unique considerations, which cannot be adequately assessed using tools developed for cohort or case–control studies. Unlike case–control studies—where participants are selected based on outcome status—or cohort studies—where they are selected based on exposure status—cross‐sectional studies typically recruit participants from an accessible population relevant to the research question. These studies do not involve prospective or retrospective follow‐ups; instead, data are collected at a single point in time to evaluate disease prevalence or explore associations between exposures and outcomes [8]. Therefore, as highlighted by a recent scoping review, the lack of a specific tool to assess RoB in cross‐sectional studies has created a gap, necessitating the development of an appropriate instrument to support researchers and readers in their evaluations [6].

The original NOS was introduced in 2009 to assess the quality of cohort and case–control studies within systematic reviews [5]. The NOS content validity, criterion validity, construct validity, and inter‐rater reliability were explored after its introduction [24]. In terms of reliability, varying degrees of inter‐rater agreement have been reported, generally ranging from low to fair [25, 26], with more favorable outcomes for test–retest agreement [27]. Overall, users have found the NOS to be more efficient and easier to apply compared to other RoB assessment tools for observational studies. However, challenges remain in scoring certain questions, which some users have found to be too vague [24, 28].

Given that the NOS scale does not include a version for evaluating cross‐sectional studies, several systematic reviews have developed *ad hoc* modifications of the NOS to assess these types of studies [6, 29, 30, 31, 32, 33]. However, the consequent proliferation of multiple, heterogeneous versions has complicated the interpretation of RoB assessments. Building on the NOS adaptation used by Romandini et al. [29] and subsequently implemented elsewhere [34], we developed the NOS‐xs as a standardized tool applicable to all cross‐sectional studies, both association and descriptive. To address the main limitations of the NOS for cohort and case–control studies, we refined the text, structure, and format of each item to enhance specificity and clarity. Additionally, to standardize the RoB assessment process, we emphasized the importance of defining key points relevant to the specific systematic review during the protocol preparation (e.g., listing the specific confounders and mediators).

As a newly proposed tool, the NOS‐xs now requires practical testing in systematic reviews. Future validation and reliability studies are also advocated, following a process similar to the development of the NOS for cohort and case–control studies. Given its strong alignment with the original tool, the content and construct validity of the NOS‐xs are, however, expected to be preserved. Future studies should specifically investigate the criterion validity of the NOS‐xs scores, as well as inter‐rater reliability and consistency in its application. Widespread adoption of the NOS‐xs in systematic reviews of cross‐sectional studies would facilitate the evaluation of these aspects, while reducing the proliferation of modified tools that introduce inconsistencies and subjectivity, thereby hindering the standardization of the RoB evaluation process.

## 7 Conclusions

When assessing the RoB of a cross‐sectional study, reviewers should focus on three main domains associated with specific biases: study sample selection (selection bias), assessment of exposure(s) and outcome(s) (information bias), and confounding factors (confounding bias). To standardize and streamline the RoB assessment process, this article introduces the NOS‐xs tool, offering researchers a unique and context‐specific instrument to evaluate the quality of cross‐sectional studies included in systematic reviews.

## Author Contributions

M.C.C. contributed to study design, and manuscript drafting. P.R. contributed to study design, and critically revised the manuscript. M.R. contributed to study conception and design, and manuscript drafting.

## Conflicts of Interest

The authors declare no conflicts of interest.

## Supporting information


Data S1.

Data S2.

Data S3.

Data S4.

Data S5.

Data S6.

Data S7.

## Data Availability

Data sharing not applicable to this article as no datasets were generated or analysed during the current study.
