# Supplementary material for: Risk of Bias Evaluation of Cross‐Sectional Studies: Adaptation of the Newcastle‐Ottawa Scale
Source: J Periodontal Res. 2025 Apr 28;61(7):837–46. doi: 10.1111/jre.13405 (PMC13665615; doi:10.1111/jre.13405)
Supplement: Supplementary file 1 [file JRE-61-837-s006.pdf]

Newcastle Ottawa Scale for cross-sectional study (NOS-xs) - ASSOCIATION STUDY			
**Study:**	Insert the reference of the original study under RoB evaluation		
**DOMAIN**	**ITEM**	**ANSWER**	**AWARDED STAR(S)**
**STUDY SAMPLE SELECTION (max. 2 stars)**	**1. Representativeness of the study sample**	◯ **a) Truly representative of the target population (all subjects or random sampling).*** ◯ **b) Somewhat representative of the target population (non-random sampling).*** ◯ c) No representative of the target population (selected group of users). ◯ d) No description of the sampling strategy.	
	**2. Sample size**	◯ **a) Justified and satisfactory.*** ◯ b) Not justified or unsatisfactory.	
**ASSESSMENT of EXPOSURE and OUTCOME (max. 4 stars)**	**3. Assessment of the exposure(s)**	◯ **a) Gold-standard assessment tool.**** ◯ **b) Acceptable assessment tool.*** ◯ c) Non-acceptable assessment methods. ◯ d) No description of the assessment tool.	
	**4. Assessment of the outcome(s)**	◯ **a) Gold-standard assessment tool.**** ◯ **b) Acceptable assessment tool.*** ◯ c) Non-acceptable assessment methods. ◯ d) No description of the assessment tool.	
**CONFOUNDING FACTORS (max. 3 stars)**	**5. Adjustment for confounder(s)**	◯ **a) The study estimates are adjusted for all the most important confounding factors, and not adjusted for potential mediator(s).**** ◯ **b) The study estimates are adjusted for all the most important confounding factors, but also for potential mediator(s).*** ◯ **c) The study estimates are adjusted for some but not all the most important confounding factors.*** ◯ d) The study estimates are not adjusted for any of the most important confounding factors or no description is provided.	
	**6. Assessment of confounder(s)**	◯ **a) Most of the confounding factors controlled for were measured through gold standard assessment methods.*** ◯ **b) Most of the confounding factors controlled for were measured through acceptable assessment methods.*** ◯ c) Most of the confounding factors controlled for were not measured through at least acceptable assessment methods. ◯ d) No confounding factors controlled for.	
	**Final Score**		
