# Supplementary material for: Risk of Bias Evaluation of Cross‐Sectional Studies: Adaptation of the Newcastle‐Ottawa Scale
Source: J Periodontal Res. 2025 Apr 28;61(7):837–46. doi: 10.1111/jre.13405 (PMC13665615; doi:10.1111/jre.13405)
Supplement: Supplementary file 2 [file JRE-61-837-s007.pdf]

Simplified Newcastle Ottawa Scale for cross-sectional study (NOS-xs2) - PREVALENCE STUDY

Study: Insert the reference of the original study under RoB evaluation

DOMAIN	ITEM	ANSWER	AWARDED STAR(S)
**STUDY SAMPLE SELECTION (max. 2 stars)**	**1. Representativeness of the study sample**	◯ **a) Truly representative of the target population (all subjects or random sampling).***<br>◯ **b) Somewhat representative of the target population (non-random sampling).***<br>◯ c) No representative of the target population (selected group of users).<br>◯ d) No description of the sampling strategy.	
	**2. Sample size**	◯ **a) Justified and satisfactory.***<br>◯ b) Not justified or unsatisfactory.	
**ASSESSMENT of OUTCOME (max. 2 stars)**	**3. Assessment of the outcome(s)**	◯ **a) Gold-standard assessment tool.****<br>◯ **b) Acceptable assessment tool.***<br>◯ c) Non-acceptable assessment methods.<br>◯ d) No description of the assessment tool.	
**Final Score**			
