# Supplementary material for: Risk of Bias Evaluation of Cross‐Sectional Studies: Adaptation of the Newcastle‐Ottawa Scale
Source: J Periodontal Res. 2025 Apr 28;61(7):837–46. doi: 10.1111/jre.13405 (PMC13665615; doi:10.1111/jre.13405)
Supplement: Supplementary file 3 [file JRE-61-837-s001.docx]

**Supplementary Material 2.** Simplified version of the NOS-xs (NOS-xs2) for cross-sectional prevalence studies.

| **Domain** | **Item** | **Answer (only 1 per item possible)** | **Rating** | **Awarded Star(s) per item** |
| --- | --- | --- | --- | --- |
| **STUDY SAMPLE SELECTION**  **(max. 2 stars)** | **1. Representativeness of the study sample** | a) Truly representative of the target population (all subjects or random sampling). | **🟑** | ___ /1 star |
|  |  | b) Somewhat representative of the target population (non-random sampling). | **🟑** |  |
|  |  | c) No representative of the target population (selected group of users). | ***-*** |  |
|  |  | d) No description of the sampling strategy. | ***-*** |  |
|  | **2. Sample size** | a) Justified and satisfactory. | **🟑** | ___ /1 star |
|  |  | b) Not justified or unsatisfactory. | ***-*** |  |
| **ASSESSMENT of OUTCOME**  **(max. 2 stars)** | **3. Assessment of the outcome(s)** | a) Gold-standard assessment tool. | **🟑🟑** | ___ / 2 stars |
|  |  | b) Acceptable assessment tool. | **🟑** |  |
|  |  | c) Non-acceptable assessment methods. | ***-*** |  |
|  |  | d) No description of the assessment tool. | **-** |  |
| **Total score** | | | | **___ / 4 stars** |
