# Supplementary material for: Risk of Bias Evaluation of Cross‐Sectional Studies: Adaptation of the Newcastle‐Ottawa Scale
Source: J Periodontal Res. 2025 Apr 28;61(7):837–46. doi: 10.1111/jre.13405 (PMC13665615; doi:10.1111/jre.13405)
Supplement: Supplementary file 4 [file JRE-61-837-s002.docx]

**Supplementary Material 1**. NOS adaptation for cross-sectional studies – association studies (NOS-xs tool).

| **Domain** | **Item** | **Answer (only 1 per item possible)** | **Rating** | **Awarded Star(s)** |
| --- | --- | --- | --- | --- |
| **STUDY SAMPLE SELECTION**  **(max. 2 stars)** | **1. Representativeness of the study sample** | a) Truly representative of the target population (all subjects or random sampling). | **🟑** | ___ /1 star |
|  |  | b) Somewhat representative of the target population (non-random sampling). | **🟑** |  |
|  |  | c) No representative of the target population (selected group of users). | ***-*** |  |
|  |  | d) No description of the sampling strategy. | ***-*** |  |
|  | **2. Sample size** | a) Justified and satisfactory. | **🟑** | ___ /1 star |
|  |  | b) Not justified or unsatisfactory. | ***-*** |  |
| **ASSESSMENT of EXPOSURE and OUTCOME**  **(max. 4 stars)** | **3. Assessment of the exposure(s)** | a) Gold-standard assessment tool. | **🟑🟑** | ___ / 2 stars |
|  |  | b) Acceptable assessment tool. | **🟑** |  |
|  |  | c) Non-acceptable assessment methods. | ***-*** |  |
|  |  | d) No description of the assessment tool. | **-** |  |
|  | **4. Assessment of the outcome(s)** | a) Gold-standard assessment tool. | **🟑🟑** | ___ / 2 stars |
|  |  | b) Acceptable assessment tool. | **🟑** |  |
|  |  | c) Non-acceptable assessment methods. | ***-*** |  |
|  |  | d) No description of the assessment tool. | **-** |  |
| **CONFOUNDING FACTORS**  **(max. 3 stars)** | **5. Adjustment for confounder(s)** | a) The study estimates are adjusted for all the most important confounding factors, and not adjusted for potential mediator(s). | **🟑🟑** | ___ / 2 stars |
|  |  | b) The study estimates are adjusted for all the most important confounding factors, but also for potential mediator(s). | **🟑** |  |
|  |  | c) The study estimates are adjusted for some but not all the most important confounding factors. | **🟑** |  |
|  |  | d) The study estimates are not adjusted for any of the most important confounding factors or no description is provided. | **-** |  |
|  | **6. Assessment of confounder(s)** | a) Most of the confounding factors controlled for were measured through gold standard assessment methods. | **🟑** | ___ / 1 star |
|  |  | b) Most of the confounding factors controlled for were measured through acceptable assessment methods. | **🟑** |  |
|  |  | c) Most of the confounding factors controlled for were not measured through at least acceptable assessment methods. | **-** |  |
|  |  | d) No confounding factors controlled for. | **-** |  |
| **Total score** | | | | **___ / 9 stars** |
