# Supplementary material for: Risk of Bias Evaluation of Cross‐Sectional Studies: Adaptation of the Newcastle‐Ottawa Scale
Source: J Periodontal Res. 2025 Apr 28;61(7):837–46. doi: 10.1111/jre.13405 (PMC13665615; doi:10.1111/jre.13405)
Supplement: Supplementary file 6 [file JRE-61-837-s005.docx]

**Supplementary Material 3. Checklist to customize the NOS-xs during systematic review protocol preparation.**

| Domain | Item | Customization Point | Customization Detail |
| --- | --- | --- | --- |
| STUDY SAMPLE SELECTION | **1. Representativeness of the study sample** | Define the target population (e.g., geographic region, specific setting). | *_(define)_* |
|  | **2. Sample size** | - | *-* |
| ASSESSMENT OF EXPOSURE AND OUTCOME | **3. Assessment of exposure(s):**  **(_______________)**  *_(Indicate the exposure)_* | Outline “gold standard” methods for exposure assessment. | *_(description)_* |
|  |  | Outline “acceptable” methods for exposure assessment. | *_(description)_* |
|  |  | Outline “non-acceptable” methods for exposure assessment. | *_(description)_* |
|  |  | Specify if calibration or blinding of exposure assessors is mandatory. | *_(calibration/blinding/none)_* |
|  | **4. Assessment of outcome(s):**  **(_______________)**  *_(Indicate the outcome)_* | Outline “gold standard” methods for outcome assessment. | *_(description)_* |
|  |  | Outline “acceptable” methods for outcome assessment. | *_(description)_* |
|  |  | Outline “non-acceptable” methods for outcome assessment. | *_(description)_* |
|  |  | Specify if calibration or blinding of outcome assessors is mandatory. | *_(calibration/blinding/none)_* |
| CONFOUNDING FACTORS | **5. Adjustment for confounder(s)** | List the “most important” confounders relevant to the review. | *_(list)_* |
|  |  | List “possible” mediators relevant to the review. | *_(list)_* |
|  |  | Specify if statistical analyses (e.g., propensity score matching) can replace confounder adjustments. | *_(yes/no)_* |
|  |  | Indicate if deviations from predefined confounders and mediators are allowed, provided they are statistically justified (e.g., “change-in-estimate” strategy). | *_(yes/no)_* |
|  | **6. Assessment of confounder(s)** | Define “gold standard” methods for assessing the “most important” confounders. | *_(description)_* |
|  |  | Define “acceptable” methods for assessing the “most important” confounders. | *_(description)_* |
